# The Impact of the Wiping Process on the Final Characteristics of Hot-Dip Galvanized Steel Wires

**DOI:** 10.3390/ma19061169

**Published:** 2026-03-17

**Authors:** Marius Tintelecan, Oscar Rodriguez-Alabanda, Ioana Monica Sas-Boca, Dana-Adriana Iluțiu-Varvara, Florin Popa, Călin-Virgiliu Prică, Ramona Pintoi

**Affiliations:** 1 Department of Materials Science and Engineering, Technical University of Cluj-Napoca, 103-105 Muncii Avenue, 400641 Cluj-Napoca, Romania; marius.tintelecan@ipm.utcluj.ro (M.T.); florin.popa@stm.utcluj.ro (F.P.); calin.prica@stm.utcluj.ro (C.-V.P.); 2 Eut+, European University of Technology, European Union, 400641 Cluj-Napoca, Romania; 3 Department of Mechanical Engineering University of Córdoba, Edificio Paraninfo, Primera Planta, Campus de Rabanales, ES-14071 Córdoba, Spain; 4 Faculty of Building Services Engineering, Technical University of Cluj-Napoca, 28 Memorandumului Street, 400114 Cluj-Napoca, Romania; 5 Department of Applied Mechanics and Civil Constructions, Faculty of Mechanics, University of Craiova, Calea București 107, 200585 Craiova, Romania; ramona.pintoi@icecon.ro; 6 ICECON Institute, Bucharest, Pantelimon Road, 266, Sector 2, 021652 Bucharest, Romania

**Keywords:** steel wires, wipe, microhardness, EDX maps, anticorrosive resistance, material circularity

## Abstract

Corrosion resistance of steel wires can be achieved through several approaches, one of the most established being hot-dip galvanizing. The effectiveness of anticorrosive protection of a galvanized wire is considered to depend not only on the galvanizing process itself, namely bath composition, temperature, and immersion duration—but also on the post-galvanizing wiping method, which ultimately determines the final thickness and uniformity of the zinc coating. This study describes and quantifies the resulting parameters of the Zn layer, systematically comparing two technical variants. Four parameters were analyzed to characterize the coating: the effective thickness of the constituent layers, their morphology (examined by SEM), their compositional profile (EDX mapping), and their microhardness. To comprehensively assess the influence of the wiping method on the anticorrosion performance of the galvanized wire, the final corrosion tests, fifth in the sequence, will be conducted in a salt fog environment using an Erichsen chamber, in accordance with standardized procedures.

## 1. Introduction

Due to continued growth of the global economy and of world population at the same time, the demand for natural resources, like fossil fuels, metals and minerals is increasing. Metals are fundamental to a circular economy, as they are infinitely recyclable without quality loss. The circularity of metals is an industrial economic model that aims instead to create a closed-loop system where metals are continuously reused, repaired, remanufactured, and recycled without losing their original properties. Because most metals can be recycled indefinitely, this approach turns waste into a valuable resource, significantly reducing the need for virgin material extraction and lowering carbon emissions and saving energy (recycling metals requires significantly less energy than producing them from virgin ore) and increasing: conservation of natural resources, environmental factors protection and sustainable development. Hot-dip galvanized steel is generally considered sustainable due to recyclability and longevity, because both zinc and steel are infinitely recyclable without compromising their quality, strength, chemical and physical properties. Galvanized steel wires have the following characteristics: durability, longevity, availability, esthetics, versatility and sustainability. Hot-dip galvanizers can contribute to the circular economy, conserving important resources and energy. Hot-dip galvanized steel wires, characterized by great resistance to corrosion in aggressive, hostile and critical environments, are crucial material for industrial (construction and building services engineering), outdoor and marine applications [1,2,3,4].

Hot-dip galvanizing is an effective corrosion protection method for steel products, such as wire, achieved by immersing the steel in a molten zinc bath at approximately 450 °C, where a strong metallurgical bond is formed. This process provides superior and long-lasting corrosion resistance, even in harsh environments. Although the initial investment may be higher than that of alternative coating methods, hot-dip galvanizing frequently proves advantageous in lifecycle cost analyses, offering over 50 years of maintenance-free protection. By eliminating the need for frequent recoating, it significantly reduces long-term costs associated with downtime, labor, and material consumption, particularly in large-scale projects or aggressive service conditions, thereby making it a superior long-term investment [5,6].

In modern industry, the addition of specific alloying elements to these zinc baths has become a critical factor in determining both the quality of the coating and the overall economics of the process. While standard galvanizing offers excellent galvanic protection, the integration of additives like aluminum (Al) and magnesium (Mg) has been the subject of intense research due to their potential to further enhance corrosion resistance. Proper management of these alloying concentrations not only limits excessive coating thickness on reactive steels and improves zinc drainage but also reduces industrial waste such as galvanizing ashes and hard zinc. However, despite clear theoretical benefits of the Zn-Al-Mg system, previous works that were consulted demonstrate that achieving widespread commercial success as a standardized industrial product remains a significant challenge [7,8,9]. Nevertheless, a pure zinc bath remains the industry standard for many general galvanizing applications due to its predictable metallurgical behavior and operational simplicity. The primary advantage lies in its ability to form an adherent, multi-layered coating consisting of distinct iron–zinc alloy phases that provide robust mechanical bonding and excellent sacrificial protection. Because the bath lacks complex ternary or quaternary alloying elements, it is significantly easier to maintain and chemically monitor, requiring less sophisticated equipment to manage concentration limits. Furthermore, pure zinc baths offer consistent “wetting” properties across a wide variety of steel grades, ensuring a uniform protective barrier without the risk of unpredictable surface defects or phase instabilities sometimes associated with experimental alloy systems. This stability translates to a highly cost-effective process for high-volume production where standardized corrosion resistance and ease of process control are prioritized.

The quality and characteristics of a galvanized coating are fundamentally shaped by the application technique, primarily distinguishing between batch hot-dip and continuous hot-dip processes. In batch hot-dip galvanizing, a part is immersed for longer periods, promoting the growth of thick iron-zinc alloy layers for maximum durability, though this can result in brittleness. In contrast, continuous hot-dip galvanizing uses high-speed immersion and mechanical wiping to produce a thinner, more uniform coating. This method suppresses brittle alloy growth in favor of a ductile zinc layer, ensuring a smooth finish and superior formability for products like wire and sheet. In this line, previous works have shown that the hot-dip process allows to obtain steel wires of both unalloyed and alloyed Zn coatings [10,11,12] and the wiping of a superficial Zn layer in the intermediate wire-drawing passes can be critical to obtain better quality of the final coated wire.

In addition to immersion time and wiping parameters, the chemical composition of the zinc bath plays a decisive role in the formation and growth of intermetallic layers. In gas-wiped continuous galvanizing, the deliberate addition of small amounts of Al promotes the formation of a thin Al–Fe–Zn inhibition layer at the steel/coating interface. This inhibition layer restricts the diffusion-controlled growth of Fe–Zn intermetallic compounds, resulting in coating structures that differ significantly from the thicker Zn–Fe intermetallic layers typically developed during conventional batch hot-dip galvanizing.

Historically, the industry relied on carbon-based media or wax-saturated wipes to regulate coating thickness; however, these methods are being replaced by gas wiping systems based on wipes made of synthetic polymers like Teflon (Polytetrafluoroethylene PTFE) as a preferred modern alternative due to their superior thermal stability, low-friction interface and cleanliness. Previous recent works assessed how the transition from traditional organic lubricants to high-performance synthetic pads affects the interfacial Fe–Zn layer morphology [13,14]. Other authors studied the performance of the gas-knife wiping technique [15] or magnetic-assisted wiping [16], demonstrating their effectiveness for the control of coating weight and thickness, but none of them included experimental comparison quantifying the advantages in product quality over the wax-saturated wiping technique or mechanical pad-/die-based systems. It should also be noted that gas wiping systems allow significantly higher processing line speeds compared with contact-based wiping methods such as pad wiping. At increased line speeds, a larger volume of molten zinc is entrained by the moving wire, which consequently requires the use of higher-performance wiping techniques (e.g., gas knives) to effectively control coating thickness and uniformity. In contrast, contact wiping systems operate at lower line speeds but offer simpler mechanical control of the retained zinc layer. In addition, these advanced techniques imply precise dynamic control of process parameters while mechanical wiping allows easy and precise dimensional control and with less zinc waste. Thus, the present study proposes and compares a simple wipe method based on wiping with non-asbestos Teflon pads in direct contact with the coated wire versus the wax-saturated wiping technique.

This work therefore presents a comparative study of two post-galvanization continuous wiping methods for coating steel wires: conventional hot-dip wiping (Technique a) and mechanical wiping via non-asbestos Teflon pads along a linear exit path (Technique b). Emphasis is placed on the mechanical wiping process, evaluating how these methods influence zinc coating thickness and, consequently, the long-term anticorrosive performance of the wire. For this, S235JR steel wires (according to EN 10025:2 [17]) with diameters of Ø 3.25 mm, Ø 2.5 mm or Ø 1.5 mm were coated, ensuring identical mechanical characteristics of the substrate wire for comparison. The final corrosion tests demonstrate that while the general hot-dip technique (Technique a) produces an iron–zinc (Fe–Zn) layer two to three times thicker than the wiped continuous process, in the case of galvanized wire wiped with non-asbestos Teflon pads (Technique b), the critical columnar alloy interface remains dimensionally unchanged. Although mechanical wiping significantly reduces the outer pure zinc layer and total coating thickness, the fundamental anticorrosive performance of the steel wire is not significantly compromised. This suggests that the wiping process, specifically using non-asbestos Teflon pads, serves as a highly efficient technical compromise and it enables a substantial reduction in zinc consumption and production costs without a major sacrifice in the wire’s protective service life.

## 2. Materials and Methods

### 2.1. Manufacturing Methodology

For a proper evaluation, three steel wire diameters will be compared: Ø 3.25 mm (samples of the two variants labeled 1a and 1b), Ø 2.5 mm (samples labeled 2a and 2b), and Ø 1.5 mm (samples labeled 3a and 3b). All three diameters were made from the same steel grade S235JR according to UNE-EN 10025-2:2019 (see Table 1) [17], ensuring identical mechanical characteristics of the steel wires for both wiping variants [7,8,9,10].

The galvanizing line was operated at a constant line speed of 50 m/min for both wiping variants. For process engineering relevance, the diameter–velocity parameter (DV = d × v) was also considered. Depending on the wire diameter, DV values ranged between approximately 70 and 200 mm·m/min. Maintaining a constant line speed ensured that the observed differences between variants were primarily associated with the wiping method rather than with variations in process kinetics.

#### 2.1.1. Technique a: Charcoal Wax Wiping

**Figure 1:**
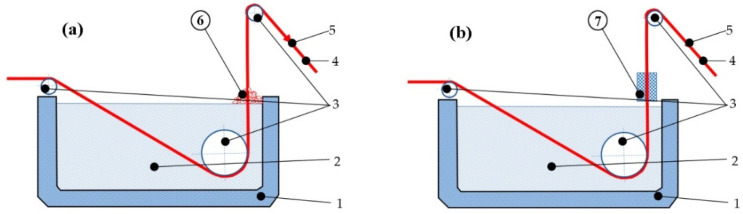
(**a**) Charcoal wax wiping of the wire after hot-dip galvanizing (technique a), (**b**) wiping the wire using two non-asbestos Teflon pads (technique b). Annotations: (1) galvanizing tub, (2) melted zinc, (3) metallic rolls, (4) steel wire, (5) pulling force, (6) charcoal wax mixture, (7) Teflon pads.

The first variant proposed consisted of immersing the steel wire in a bath of molten Zn (at 450 °C) for about 1.5 min, followed by wiping the surface of the wire with a pile of charcoal wax mixture while the steel wire is being pulled in a continuous operation as shown in Figure 1a.

#### 2.1.2. Technique b: Wiping with Non-Asbestos Teflon Pads

The proposed and analyzed technique involves wiping the steel wire after identical galvanization by immersion in a molten zinc bath (at the same temperature of 450 °C and the same immersion time of 1.5 min), but using two non-asbestos Teflon pads, pressed onto the surface of the wire at the exit of the system (see Figure 1b).

Bath temperature has been set to 450 °C and the immersion time to 90 s. The hot-dip galvanizing process was performed using a molten zinc bath with a purity level of at least 99,995% by weight, in full compliance with the requirements specified in the EN 1179 standard [6], as indicated in Table 2.

### 2.2. Sample Wire Material

All samples (1a, 2a, 3a, 1b, 2b, and 3b) were made from the same steel grade, S235JR according to UNE-EN 10025-2:2019 [17]. The chemical composition of this steel is shown in Table 1 [16].

In the deposition process, initial Zn was used in molds, according to standard EN 1179:2003 [6].

To ensure that the results were not influenced by variations in the steel wire surface, the experimental methodology utilized an identical preparation route for both technical variants. This process began with in-line annealing to stabilize the mechanical properties and thermally clean the wire, followed by a cooling in water and chemical pickling in a 20% hydrochloric acid solution at room temperature (≈23 °C) to remove oxides and achieve a chemically active surface. After the HCl pickling step, the steel wires were thoroughly rinsed with water prior to immersion in the molten zinc bath in order to remove any residual acid and avoid contamination of the galvanizing bath. By standardizing these pretreatment stages, the study effectively isolates the galvanizing and wiping techniques as the sole variables affecting the iron–zinc (Fe–Zn) layer formation, ensuring that any differences in coating quality are due to the deposition method rather than surface inconsistencies.

### 2.3. Coating Characterization, Equipment and Procedures

**Figure 2:**
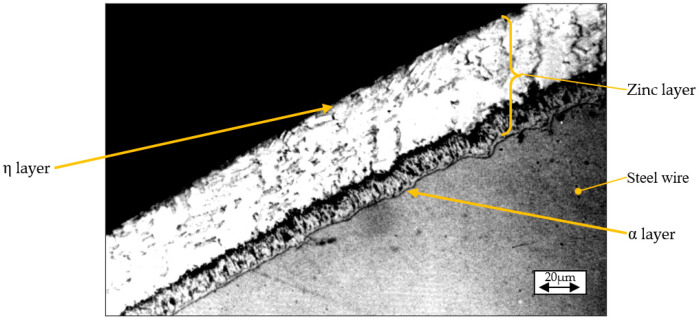
The microstructure of the zinc layer deposited on the steel wire and the main sub-layers (phases) identified.

According to the literature, the final zinc layer produced by thermal galvanizing followed by light smoothing consists of some successive sublayers/phases (α, γ, δ1, δ, ξ, and η) whose characteristics are summarized in Table 3 and which are in correspondence with those phases that have been identified in previous works consulted [7,18,19,20,21]. The aspect of them, recorded by optical microscopy, is shown in Figure 2.

The thickness of the deposited zinc layer was considered as a determining indicator and was used to establish a quantitative basis for comparing the two technical variants under study. Following the specification of UNE-EN ISO 1460:2021 standard [18], a gravimetric analysis was performed, which involves weighing the samples before and after the chemical removal of the coating to calculate the mass of zinc per unit area which involves weighing the samples before and after the chemical removal of the coating using a 5% hydrochloric acid (HCl) solution at room temperature to calculate the mass of zinc per unit area. This process yielded six distinct numerical values corresponding to the mass per unit area (*m_t_*) expressed in g/m^2^ and coating thickness (*t*) in µm for all of six specimen samples (1a, 2a, 3a, 1b, 2b, 3b) constituting the experimental setup. To ensure statistical reliability and to consider any local variations in the coating, the average coating thickness *m_t_* was calculated as the arithmetic mean followed by the standard deviation of five individual measurements made in different portions of each single wire (see Table 4).

Additionally, to ensure the accuracy of the measurement of this parameter, validation measurements were performed directly on the images obtained by SEM.

The compositional profiles and EDX maps of each sample have been systematically analyzed using JEOL JSM-5600LV scanning electron microscope (JEOL Ltd., Tokyo, Japan), allowing the comparison between results obtained for each diameter and wiping technique combination. In addition, radial microhardness of the zinc layer (HV) has been measured using AFFRI DM8-B high precision, automated Vickers microhardness testing system (AFFRI Testing Instruments, Varese, Italy). Measurements were performed in 5 areas around the coated section of each sample. A Vickers indenter with a 50 g-f load (HV0.05) was employed. Considering that the thickness of individual coating sublayers ranges from approximately 15 µm to 70 µm (see Section 3.3), this indenter size is sufficiently small to provide localized hardness measurements within each phase without averaging across multiple layers. For the SEM and microhardness, the samples were resin-embedded and polished for achieving a flat surface.

In this line, the comparative characterization has been complemented by a detailed identification of the constitutive phases (sublayers) based on the images obtained after the hot-dipping process by both wiping techniques. This has been done by analyzing the compositional profiles obtained by SEM and EDX maps and measuring the microhardness values localized in the different identified sublayers (phases) of the coating.

To evaluate the corrosion resistance of the coatings (for both types of coating techniques), an Erichsen salt spray chamber was used, in accordance with the UNE-EN ISO 9227:2023 [22] standard (equivalent to ASTM B117). This accelerated testing involves exposing the samples to a continuous atomized fog of 5% ± 1% sodium chloride solution at a controlled chamber temperature of 35 °C ± 2 °C and a pH range of 6.5 to 7.2. Thus, the procedure provides a consistent corrosive environment to accurately compare the protective performance and time to white and red rust formation, identically, for the studied techniques. The test has been repeated five times to obtain the time (in hours) until white rust (early oxidation) and red rust (full oxidation) appearance.

**Figure 3:**
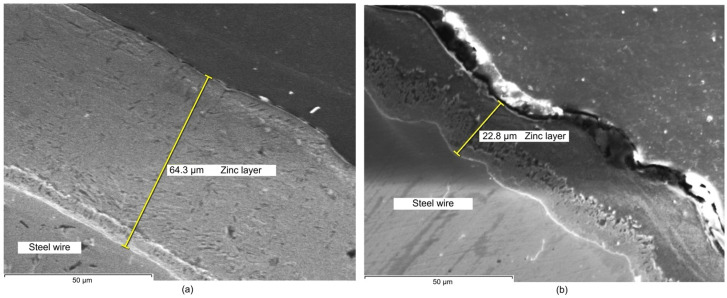
SEM images of the zinc layer deposited on the steel wire: (**a**) corresponding to sample 1a and (**b**) to sample 1b.

**Figure 4:**
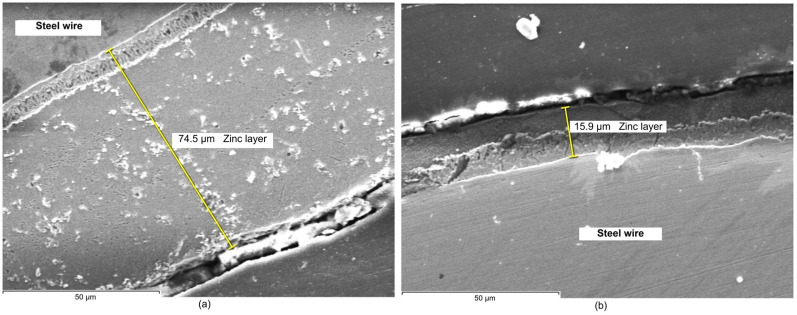
SEM images of the zinc layer deposited on the steel wire: (**a**) corresponding to sample 2a and (**b**) to sample 2b.

**Figure 5:**
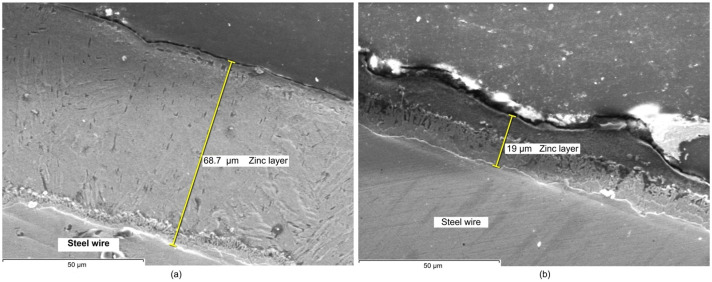
SEM images of the zinc layer deposited on the steel wire: (**a**) corresponding to sample 3a and (**b**) to sample 3b.

**Figure 6:**
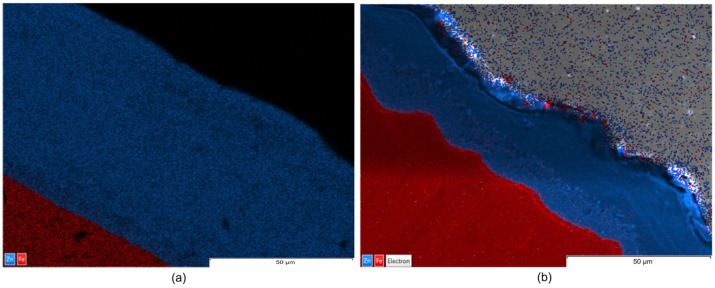
EDX maps: (**a**) corresponding to sample 1a and (**b**) to sample 1b.

**Figure 7:**
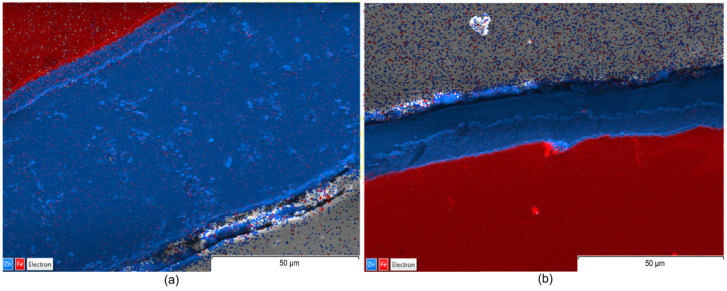
EDX maps: (**a**) corresponding to sample 2a and (**b**) to sample 2b.

**Figure 8:**
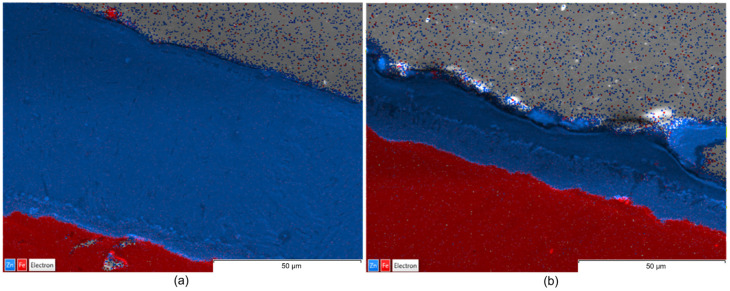
EDX maps: (**a**) corresponding to sample 3a and (**b**) to sample 3b.

**Figure 9:**
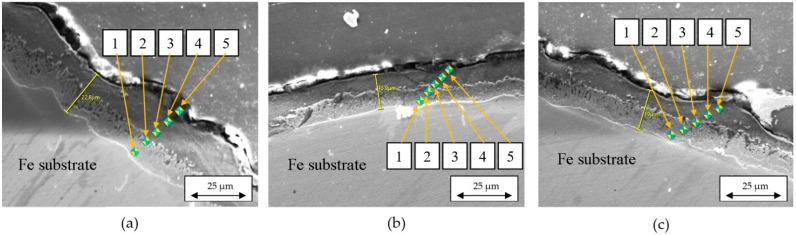
Location of the control points where microhardness was measured: (**a**) corresponding to sample 1b, (**b**) sample 2b, and (**c**) sample 3b.

**Figure 10:**
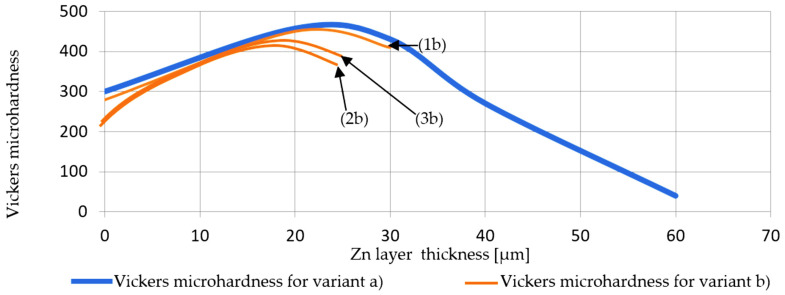
Vickers HV_0.5_ microhardness evolution as a function of the layer thickness: comparison between evolution in samples made by Teflon pads wiping method (technique b) vs. mean microhardness evolution in the samples made by wax-wiping method (technique a); 1b represents the 3.25 mm diameter steel wire protected with the b protection technique; 2b is the 2.50 mm diameter steel wire protected against corrosion with the b technique; 3b is the 1.50 mm diameter steel wire, protected against corrosion with the b technique.

**Figure 11:**
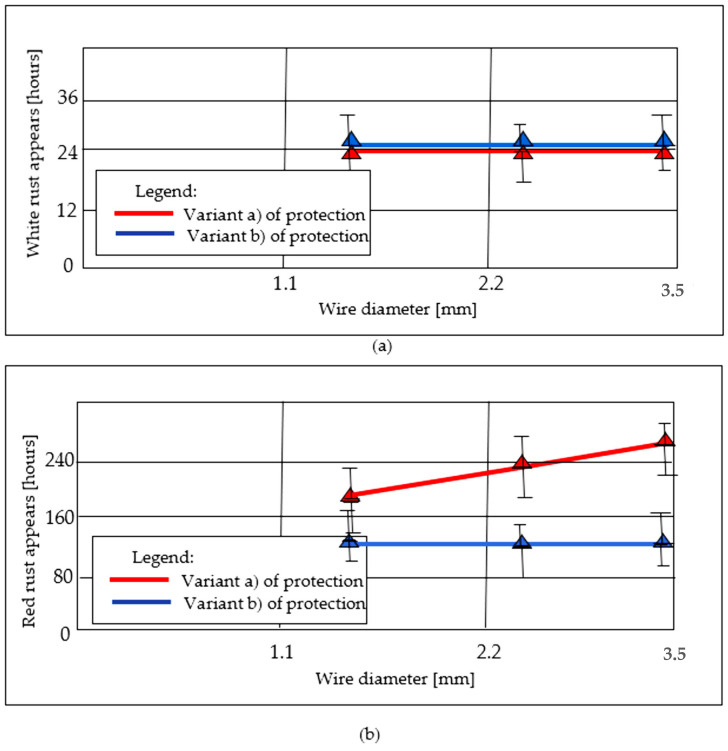
Duration [hours] of the corrosion process until (**a**) the appearance of white rust, (**b**) the appearance of red rust.

Summarizing, the specific objectives and the results identification (in parenthesis) are as follows: (i) quantifying the thickness of the deposited zinc layer (Table 4), (ii) obtaining compositional profiles of the deposited layers for all six samples (Figure 3, Figure 4 and Figure 5), (iii) determining the elemental distribution of Zn and Fe within the same layers by EDX mapping (Figure 6, Figure 7 and Figure 8), (iv) measuring the microhardness at various points within the Fe and Zn layers according to UNE-EN ISO 6507-1:2024 [23] (with measurement locations shown in Figure 9 and results in Table 5 and Figure 10), and (v) evaluating the corrosion resistance of coatings by the Erichsen method according to UNE-EN ISO 9227:2023 [22] (Table 6 and Figure 11).

The main objective of this work is to evaluate whether aggressive mechanical wiping—specifically using non-asbestos Teflon pads—can significantly optimize zinc consumption and process costs without compromising the fundamental anticorrosive performance of the wire. By analyzing the relationship between the reduced pure zinc layer and the stable iron–zinc (Fe–Zn) alloy interface, this research seeks to validate a more technically efficient wiping variant for its industrial application in galvanizing.

According to Table 4 on page 6 of the article: 1b represents the 3.25 mm diameter steel wire protected with the b protection technique (wiping with those Teflon pads pressed onto the surface of the wire), 2b is the 2.50 mm diameter steel wire protected against corrosion with the b technique and 3b is the 1.50 mm diameter steel wire, protected against corrosion with the b technique. Technique a was the classic one (wiping with charcoal-wax) and technique b was wiping with Teflon pads (plates) pressed onto the surface of the wire.

## 3. Results and Discussion

### 3.1. Determination of the Zn Deposited Layer Thickness

The initial step consisted of accurately determining the thickness of the thermally deposited Zn layer for each wiping technique. For this, the average thickness value of the Zn coating (*t*) has been obtained from conversion of coating mass (*m_t_*), in g/m^2^, to coating average thickness, in µm, according to the UNE-EN ISO 1460:2021 standard [18]. Complementarily, these values were validated by direct measuring in the SEM images. The results are summarized in Table 4, where RSD is the Relative Standard Deviation:

The experimental results provided in Table 4 demonstrates that the characteristics of the hot-dip galvanized coating are significantly influenced by the wiping technology employed, with the wire diameter acting as a secondary, though notable, factor in coating distribution. Thus, the primary driver of coating thickness (*m_t_*) is the wiping method, which created two distinct product classes. In addition to the average thickness values, the angular uniformity of the coating around the wire circumference was evaluated. For both wiping techniques, measurements at multiple angular positions indicated that wax wiping (technique a) produced slightly more variable thickness around the wire, with minor peaks and troughs due to the less controlled drag-out of molten zinc. In contrast, Teflon pad wiping (technique b) yielded a more concentric coating, showing higher uniformity in the angular direction. This confirms that the mechanical action of the pads not only reduces the total coating thickness but also improves the circumferential homogeneity of the Zn layer. Technique (a), wax wiping, functioned as a passive process that allowed for heavy zinc accumulation, resulting in thick coatings (*t* ≈ 61 µm to 71 µm) suitable for high-corrosion environments. These thickness values and homogeneity of Zn and Zn-alloy galvanized steel coatings are in correspondence with those obtained by direct measurement methods in previous works consulted [24], although Verma et al. obtained thicker Zn coatings but increased the dipping time to 3 min [25]. In contrast, technique (b) utilized Teflon pads as a mechanical “squeegee” to actively strip away excess molten zinc, which has produced a much thinner, more uniform layer (*t* ≈ 15 µm to 27 µm) that optimizes material usage and improves surface finish. Although this improvement is significant, previous works demonstrate that implementing more sophisticated wiping systems such as air-knife wiping [26] is possible in order to get better homogeneity in the coating layer with variations of less than 2 µm in coatings of ≈ 53 µm. Nevertheless, the values obtained with the Teflon pads wiping system (option b) is in correspondence with those obtained by Wang [27], who obtained Zn coating thickness values between 40 and 120 µm using the air-knife wiping system.

While the wiping method set the general thickness range, the wire diameter introduced non-linear variations maybe due to complex fluid dynamics at the exit of the zinc bath. The data has suggested a “critical point” at the Ø2.50 mm diameter, which produced both the thickest results in the wax process and the thinnest in the Teflon process. This indicates that surface tension and the mechanical efficiency of the pads were highly sensitive to the curvature of the wire, requiring precise calibration to ensure uniformity across different gauges.

On the other hand, the results confirmed a high correlation between gravimetric mass calculations and SEM imaging validation measurements. The calculated thickness (*t*) and validated thickness (*t_v_*) have typically aligned (RSD being within 1% to 5%), adhering to the UNE-EN ISO 1460:2021 standard [18]. The slight tendency for SEM values to exceed gravimetric averages of thickness (*t*) in wax-wiped samples can be due to local surface irregularities and the presence of a thicker alloy layer, which could be better captured by direct measuring in microscopic cross-sections than in a global mass average calculation.

From a technical and industrial perspective, these results highlight a trade-off between durability and formability. Wax wiping (option a) maximized the cathodic protection lifespan but may be more prone to flaking during intense mechanical deformation. Teflon pad wiping (option b) can provide a highly adherent, smooth coating that is ideal for wires requiring further drawing or bending, thereby offering a superior balance of dimensional precision and cost-effective zinc consumption. Furthermore, the drastic reduction in thickness using Teflon pads resulted from a shift from passive fluid dynamics to active mechanical stripping. Thus, the wax method has been characterized by “drag-out” volume, while the pad method was defined by mechanical wiping.

Considering the lower thickness values obtained by Teflon pad wiping (option b) in the present study, they are estimated to last between 10 and 20 years under relatively mild conditions [28], and for compensation for these lower thicknesses in practice, several process variables need to be adjusted to increase zinc retention. Reducing the clamping pressure of the pads or increasing the wire speed can allow for obtaining a thicker film of zinc. Additionally, a slight lowering of the bath temperature increases the viscosity of the molten zinc, making it more resistant to the stripping action of the pads. These calibrations can enable the achievement of greater coating thickness while maintaining the coating surface smoothness and thickness homogeneity provided by this mechanical wiping.

### 3.2. Microstructural Caracterisation by SEM and EDX Mapping of the Deposited Zn Layer

The resulting SEM images corresponding to the zinc coating layer obtained in the experiments are shown in Figure 3, Figure 4 and Figure 5. As can be seen, images allow us to appreciate the dimension and homogeneity of the coating layers.

The microstructural analysis of the zinc coating layers observed in SEM images in Figure 3, Figure 4 and Figure 5 reveals a clear correlation between the wiping method and the resulting layer characteristics. In terms of dimension, samples processed with wax wiping (technique a, Figure 3a, Figure 4a and Figure 5a) consistently exhibit a significantly higher coating thickness, ranging from approximately 64 µm to 74 µm. Conversely, the use of Teflon pads (technique b, Figure 3b, Figure 4b and Figure 5b) results in a substantial reduction in layer thickness, yielding dimensions between approximately 15.9 µm and 23 µm. These values validate the results obtained by gravimetric methodology, indicating that Teflon pads exert a more restrictive mechanical action, effectively removing excess molten zinc more efficiently than the wax-wiping technique. Regarding homogeneity in thickness, SEM micrographs reveal that both methods produce continuous and compact layers. However, the wax-wiped samples generally maintain a patchy physical appearance and thickness of the Zn layer, while the Teflon-wiped samples achieve a thinner, more refined coating but they exhibit more pronounced thickness irregularities, as can be seen in Figure 3b and Figure 5b. Neither of the analyzed samples induced chemical stratification, not even within the zinc-rich alloy sub-layer identified in contact with the Fe substrate interface in the SEM images.

Complementarily, EDX maps illustrate the elemental distribution and interfacial chemistry of the coating layers (Figure 6, Figure 7 and Figure 8).

The EDX maps illustrate that, although Teflon pads were used and the coating size was significantly reduced, the chemical homogeneity and purity of the zinc layer is maintained as effectively as the traditional wax-wiping method. This suggest that Teflon pad wiping is a viable alternative for achieving thinner, high-quality, and chemically uniform Zn coatings without the surface defects typically associated with high-pressure gas wiping. Images in Figure 6, Figure 7 and Figure 8 identify a uniform Zn–Fe interfacial line, confirming that both wiping methods produced chemically uniform zinc layers with a strong metallurgical bond to the steel core, verifying that the mechanical reduction in thickness does not compromise the coating’s elemental integrity or adhesion.

From a comparative point of view, EDX maps of element distribution combined with SEM revealed that, while wax-wiping resulted in significantly thicker coatings (~60–74 µm, considering all measured values), as a robust protective barrier of pure zinc, Teflon pad wiping successfully produced much thinner layers (~15–27.5 µm, considering all measured values) but without compromising chemical purity. A key distinction observed in the Teflon-wiped samples has been a sharper, more defined interface between the zinc and the steel core, likely resulting from the mechanical compression and rapid cooling inherent to the pad-wiping process. Ultimately, both methods ensure elemental integrity of the Zn coating, but Teflon pads offer a more distinct chemical boundary alongside their superior dimensional control. In addition, highly compact coatings free of internal voids, porosity, or foreign inclusions have been obtained in all cases.

The consistent color intensity across the EDX maps verified the predomination of zinc concentration even in the transition sub-layer in contact with Fe, demonstrating that none of the processes caused chemical stratification, not even in the Zn-rich alloy sub-layer in contact with the substrate that could be identified in the SEM images. Nevertheless, a detailed examination of the SEM images (Figure 3, Figure 4 and Figure 5) reveals material diffusion in the intermetallic region characterized by the formation of Fe–Zn alloy phases resulting from the metallurgical reaction during the hot-dip process. While the total coating thickness decreases notably in Teflon-wiped samples, the thickness of this diffusion sub-layer remains relatively uniform across all specimens. In addition, this intermetallic transition layer appears proportionally thicker in variant b (Teflon wiping) compared to variant a (wax wiping). This phenomenon can be explained because the Teflon pads exert a more restrictive mechanical action that strips away a significant portion of the outer “free” zinc, mechanically compacting the remains over the Fe substrate. Consequently, the alloy sub-layer occupies a much larger percentage of the total cross-section in variant b, making the metallurgical transition appear more prominent and the chemical boundary appear sharper in SEM and EDX analysis. These observations are compatible with the typical morphology found for a pure Zn hot-dipped coating [23], and the effect obtained with Teflon pad mechanical wiping are in correspondence with results shown in previous consulted work developed by Suliga et al. [29] in which a C42D steel wire passes through successive drawing dies. In this case, mechanical action of the drawing die makes the coating thinner, but diffusion, as well as phase remodeling of individual structural components, occurs since the forming process happens with a solid layer of Zn.

### 3.3. Determination of Microhardness Distribution in the Coated Samples

Mechanical properties of the zinc coatings were characterized by correlating their microstructural features with local Vickers microhardness values. Optical microscopy was employed to precisely document the indentation sites across the coating’s cross-section (Figure 9), ensuring that measurements accurately represented the distinct metallurgical zones identified in the previous SEM analysis. Microhardness was measured for each of the five points as shown in Figure 9a–c, on five areas around the coating section in each sample. The resulting data illustrates the distinct evolution of mechanical resistance across the thickness of the galvanized layers and is detailed in Table 5 and synthesized in Figure 10.

Figure 10 illustrates the numerical values in graphical form.

Analysis of the microhardness profiles reveals that the wiping-induced variations in thickness and interfacial sharpness significantly influenced the coating’s structural integrity and deformation resistance. For the Teflon-wiped samples (variant b), HV values generally increased from values between 226 and 288 HV near the substrate interface toward the center of the coating, reaching peak values between 406.7 and 434.1 HV before slightly tapering off (Table 4). These results are in line with the work by Kiełpińska et al. [30], in which they galvanized S235 steel flat samples by immersion, demonstrating that the HV value is directly affected by galvanizing time. After 90 s of galvanizing, the highest microhardness of the coating they recorded for S235 steel was 271.2 HV for a thickness of ~90 µm, a value notably lower than those obtained in the present work even at lower galvanizing time and with notably lower coating thickness mechanically compacted by wiping with Teflon pads. In another recent work, Szatkowska et al. [31] obtained Zn coating microhardness between 300 and 400 HV by the post-application of a heat treatment in a galvanized steel flat sample of DC01 low-carbon steel, maximum values very similar to those that have been obtained using the Teflon pads mechanical wiping proposed in this work.

When comparing the two methods (variant a and b), graphs in Figure 10 show that while both variants followed a similar trend, the Teflon-wiped samples achieved a more compact distribution of HV within a more compact thickness range than the wax-wiped counterparts (variant a). This observation confirms that the mechanical reduction in layer thickness through Teflon pads does not compromise the hardness properties of the coating; rather, it provides a consistent hardness profile that confirms the high structural homogeneity and successful metallurgical bonding of the refined zinc layer.

### 3.4. Study of Corrosion Resistance

The corrosion resistance of the galvanized samples was evaluated through saline fog testing on an Erichsen installation to determine the onset of oxidation for both the protective coating and the base metal. The results are shown in Table 6.

Figure 11 illustrates the evolution of rush appearance as a function of time and wire diameter in each of cases that are an object of this study.

In Figure 11, to further illustrate the effect of the wiping process on the coating, cross-section images before and after corrosion are provided to show the Zn layer morphology and potential changes in coating weight for both wiping techniques. Additionally, weight-loss measurements using the weigh–strip–weigh method are included for both uncorroded and corroded samples for completeness. No significant differences in overall coating integrity were observed between the two wiping methods. As can be seen, the time to appearance of white rust, which marks the initial oxidation of the zinc layer (primarily ZnO), remained consistent at 24 h across all samples. This indicates that the initial chemical stability of the zinc surface is independent of both the wire diameter and the specific wiping method employed. In contrast, a significant divergence was observed in the time to appearance of red rust, representing the critical moment of base metal oxidation. The red coloration observed in the corroded samples may result not only from steel attack but also from the corrosion of the Fe–Zn intermetallic compounds. This distinction is important to accurately interpret the early stages of corrosion in the coating. For samples processed with the wax-wiping method (variant a), the resistance time showed a sharp positive correlation with increasing wire diameter, reaching up to 264 h for the largest diameter sample (1a). Conversely, the alternative Teflon pad wiping method (variant b) exhibited a near-constant resistance duration of approximately 120 h, regardless of the wire diameter. This behavior suggests that while the wax-wiping method provides superior long-term sacrificial protection proportional to the resulting coating thickness, the Teflon pad wiping method maintains a consistent, shorter, protective lifespan due to its highly uniform and dimensionally controlled refined layer. Many previous works corroborate the high impact of the thickness parameter on the corrosion resistance of Zn-coated steels [32]. When the time to appearance of red rust is considered as the moment of base metal oxidation, a noticeable difference appears between the two protection options: namely, a sharp increase in this time with increasing wire diameter for option a (wax wiping), and a near-constant duration regardless of wire diameter for the alternative protection variant b (Teflon pads mechanical wiping). These results evidence that, although coating microhardness increased, the formation of the Fe–Zn intermetallic phase worsens corrosion resistance, as evidenced in previous works that have been consulted [33]. Recent research confirmed industrial interest in the development of new methods to improve the galvanizing process to obtain protected steel wires with low thicknesses around 15 µm to 30 µm [34,35,36]. In this sense, Barmatov et al. [36] evaluated the corrosion behavior of galvanized steel wires galvanized with 15 µm thickness under accelerated atmospheric corrosion conditions created by a solution of 3 wt% NaCl, concluding also that the corrosion front does not penetrate into the steel core of the wires during the 24 h corrosion test. Furthermore, the analyzed wires showed good performance against long-term corrosion.

## 4. Conclusions

Based on the integrated results from microstructural analysis, microhardness testing, and corrosion resistance analysis, several clear relationships are evidenced between coating thickness, hardness, and corrosion performance.

The integrated analysis confirms a direct positive correlation between coating thickness and long-term corrosion resistance, particularly regarding the time to base metal oxidation (red rust). Samples processed with wax wiping (variant a) produced significantly thicker layers, which resulted in superior sacrificial protection that increased proportionally with wire diameter, reaching up to 264 h. In contrast, the thinner, more refined coatings produced by Teflon wiping (variant b) exhibited a constant resistance of approximately 120 h regardless of diameter, establishing that the total volume of zinc remains the primary determinant for the duration of substrate protection.

Regarding mechanical properties, coating thickness dictates the distribution of Vickers microhardness (HV) across the galvanized layer. While both wiping methods show a similar trend where hardness increases from the substrate interface toward the coating center, the Teflon-wiped samples achieve peak hardness values up to 434.1 HV within a compact thickness range. This demonstrates that the mechanical action of Teflon pads creates a more compact and strong coating layer without compromising the structural integrity or the metallurgical bond with the substrate.

Finally, the results indicate that the initial onset of corrosion is independent of the coating’s mechanical and dimensional characteristics. The time to the appearance of white rust, marking the initial oxidation of the zinc surface, remained constant at 24 h for all tested samples. This suggests that the initial chemical reactivity and surface stability of the zinc are unaffected by the differences in peak hardness or the total thickness achieved through the various wiping processes.

Specifically, the main conclusion of the results obtained in the present study can be summarized as follows:

- The thickness data obtained (Table 4) demonstrate that the Zn coating thickness obtained by wax wiping (variant a) is approximately 2–3 times greater than that with mechanical Teflon pads wiping (variant b).
- Although the total layer thickness is strongly affected in wiping variant b, the transition layer near the Fe–Zn remains, as evidenced by the SEM micrographs in Figure 3, Figure 4 and Figure 5.
- The evolution of microhardness (from the graph in Figure 10) shows that the wiping mode of variant b primarily affects the outer layer of pure Zn, while overall anticorrosive protection remains slightly affected.
- Although coating thickness, EDX mapping, and microhardness of the layer in variant a (wax wiping) are significantly altered compared to technical variant b (Teflon pads wiping), the corrosion resistance of the steel wires does not fundamentally differ between the two wiping variants.
- Compositional analysis and EDX mapping indicate no major technical differences depending on the wire diameter, confirming the feasibility of the Teflon pad wiping for this range of wire sizes.
- As a corollary, the implementation of the Teflon pad wiping system is postulated as a technically worthy and industrially viable alternative to traditional wax methods. While wax wiping provides a larger sacrificial zinc reservoir, the Teflon-based approach ensures exceptional dimensional precision, structural homogeneity, and a more compact microhardness profile without compromising chemical purity. Given the industrial demand for high-precision coatings that optimize raw material consumption while maintaining metallurgical integrity, this innovation could significantly enhance process control and operational efficiency with a simple wiping system. The implementation of Teflon pads offers a simple, cheap and feasible solution for the adjustment of coating quality in large-scale steel wire production, balancing refined coating performance with substantial potential for cost-effective manufacturing.

## Figures and Tables

**Table 1:** Chemical composition of the S235JR steel used for the tested samples. Fe content not given as balance because the standard does not specify the full elemental composition.

C	Mn	S	P	N	Cu	Component
max. 0.17	max. 1.4	max. 0.35	max. 0.35	max. 0.012	max. 0.55	Concentration [wt%]

**Table 2:** The chemical composition of the Zn used, the quality being called ZnZ1.

Zn	Pb	Cd	Fe	Cu	Sn	Component
Min. 99.995	max. 0.003	max. 0.003	max. 0.002	max. 0.002	max. 0.002	Concentration [wt%]

**Table 3:** Characteristics of the constituent layers of the deposited zinc coating.

Layer (Phase)	Chemical Formulae	Iron Concentration [wt%]	Typical Microhardness [HV]
α	Fe–Zn	80–100	max. 300
γ	Fe–Zn	55–100	-
δ_1_	FeZn_7_	7.0–11.5	avg. 457 (427–545)
δ	FeZn_7_	7.0–10.0	-
ξ	FeZn_15_	6.0–6.2	avg. 270 (200–427)
η	Zn	max. 0.003	37–40

**Table 4:** Thickness of Zn layer on the specimens: calculated, from SEM and by direct measuring.

Tested Sample	Steel Wire Diameter, *d* [mm]	Technical Variant of Protection	Mass Per Unit Area, *m_t_* [g/m^2^]	Avg. Coating Thickness, *t* [μm] ± RSD [%]	SEM Validated Coating Thickness, *t_v_* [μm]
1a	Ø 3.25	a	434.10	60.9 ± 4.7	64.3
2a	Ø 2.50	a	510.17	71.5 ± 3.1	74.2
3a	Ø 1.50	a	465.73	65.2 ± 3.6	68.7
1b	Ø 3.25	b	195.74	27.4 ± 4.6	22.8
2b	Ø 2.50	b	107.07	15.0 ± 2.4	15.9
3b	Ø 1.50	b	125.33	17.5 ± 3.2	19.0

Note that mass per unit area (*m_t_*) throws calculated thickness (*t*) for a zinc density of 7.14 g/cm^3^, according to UNE-EN ISO 1460:2021 [18].

**Table 5:** HV microhardness average values in the samples galvanized by Teflon pads wiping.

Sample	1	2	3	4	5
1b	288.2 ± 7.2	350.5 ± 7.1	415.5 ± 8.1	434.1 ± 9.0	408.5 ± 7.7
2b	226.8 ± 4.3	327.0 ± 5.3	406.7 ± 3.3	371.9 ± 6.1	-
3b	237.0 ± 6.1	338.7 ± 5.7	411.6 ± 4.5	385.8 ± 6.2	-

**Table 6:** Corrosion exposure times until rust appearance in the galvanized samples.

	Samples					
	1a	1b	2a	2b	3a	3b
Time to appearance of white rust [hours]	24 ± 4.1	24 ± 6.0	24 ± 4.7	24 ± 4.4	24 ± 5.2	24 ± 5.5
Time to appearance of red rust [hours]	264 ± 16.5	120 ± 21.5	192 ± 20.1	120 ± 23.4	168 ± 17.8	120 ± 16.1

## Data Availability

The original contributions presented in this study are included in the article. Further inquiries can be directed to the corresponding authors.
